# Covert vascular brain injury in chronic kidney disease

**DOI:** 10.3389/fneur.2022.824503

**Published:** 2022-07-25

**Authors:** Kaori Miwa, Kazunori Toyoda

**Affiliations:** Department of Cerebrovascular Medicine, National Cerebral and Cardiovascular Center, Suita, Japan

**Keywords:** chronic kidney disease, albuminuria, cerebral small vessel diseases, brain, stroke

## Abstract

Chronic kidney disease (CKD) contributes to the increased risk of stroke and dementia. Accumulating evidence indicates that structural brain abnormalities, such as cerebral small vessel disease, including white matter hyperintensities, lacunes, perivascular spaces, and cerebral microbleeds, as well as brain atrophy, are common in patients with CKD. All of these imaging findings have been implicated in the development of stroke and dementia. The brain and kidney exhibit similar impairments and promote structural brain abnormalities due to shared vascular risk factors and similar anatomical and physiological susceptibility to vascular injury in patients with CKD. This indicates that kidney function has a significant effect on brain aging. However, as most results are derived from cross-sectional observational studies, the exact pathophysiology of structural brain abnormalities in CKD remains unclear. The early detection of structural brain abnormalities in CKD in the asymptomatic or subclinical phase (covert) should enable stroke risk prediction and guide clinicians on more targeted interventions to prevent stroke in patients with CKD. This article summarizes the currently available clinical evidence linking covert vascular brain injuries with CKD.

## Introduction

Chronic kidney disease (CKD) affects 9.1% of the global population (1). Patients with CKD have an increased burden of cardiovascular disease, and the risk of dying from a cardiovascular event is greater than the risk of end-stage renal disease (ESRD). CKD has continued to rise in rank among the leading causes of death in relation to aging and increased burden of vascular risk factors (1). Stroke is the leading cause of death in patients with CKD worldwide, and is associated with a 5-fold increase in stroke, and even mild reductions in glomerular filtration rate (GFR) are associated with substantial increase in the risk of stroke (2, 3). Moreover, CKD is an independent high-risk factor for neurological deterioration, disability, and mortality after stroke (4–6). Given the overwhelming clinical impact of CKD, it is important to evaluate kidney dysfunction to predict CKD progression. GFR and albuminuria are used to classify CKD; GFR is a marker of renal excretory function, while albuminuria is an indicator of renal barrier dysfunction.

The increased incidence of stroke in CKD is not only due to the aging process (7), but also due to the high prevalence of vascular risk factors, including hypertension and diabetes, and CKD-induced factors, such as hyperuricemia and anemia. Particularly, CKD increases vascular dysfunction and accelerates endothelial dysfunction, arterial media stiffness, and media calcification, which in turn increases the risk of stroke (8). Even mild CKD accelerates endothelial dysfunction and promotes vascular stiffness due to changes in the proin?ammatory and pro-thrombotic microenvironment (9). Specifically, abnormalities in the kidney measurements can be associated with a chronic proinflammatory state, which may accelerate microvascular damage by endothelial dysfunction, resulting in blood-brain barrier (BBB) dysfunction and causing microglial activation with subsequent neuronal injuries (10).

Thus, apart from clinically overt stroke, there is an increasing interest in understanding the coexistence of decreased GFR or increased albuminuria and brain structural abnormalities. Adaptive changes in brain structural abnormalities include cerebral small-vessel disease (SVD) and large-vessel disease, all of which lead to an increased risk of overt stroke and dementia with aging (11–14). Early detection of structural brain abnormalities in CKD and ESRD in the asymptomatic or subclinical phase (covert) should provide crucial insights into the pathobiology of CKD, improve stroke risk prediction, and guide clinicians regarding better-targeted interventions to prevent stroke in patients with CKD. We aimed to provide a narrative overview of the main clinical manifestations of covert vascular brain injury and its pathologies in patients with CKD. This review discusses magnetic resonance imaging (MRI) markers of SVD, including white matter hyperintensities (WMHs) of presumed vascular origin, lacunes, perivascular spaces (PVSs), cerebral microbleeds (CMBs), intracranial atherosclerotic stenosis, microstructural changes in the white matter, brain atrophy, and impaired cerebral blood flow (CBF) in patients with CKD.

## Pathogenesis of vascular abnormalities in renal impairment

The kidney and brain share similar microvasculature and vasoregulation, leading to shared susceptibility to microvascular dysfunction. They are both low-resistance end organs that are continuously exposed to high-volume blood flow (15 and 20% of resting cardiac output, respectively) and fluctuations in pressure, and they have local autoregulation. Although the kidneys are relatively small and account for only 1% of the total body weight, they have twice the oxygen consumption of the brain and receive a 7-fold higher blood flow than that of the brain under resting conditions (10).

Brain arterioles arising from perforating arteries are morphologically similar to kidney juxtamedullary arterioles, and both are responsible for maintaining a strong vascular tone, leading to a sufficient pressure gradient from parent vessels to capillaries (15). The perforating vessels in the brain and the afferent arterioles of the glomerulus are short in length and are exposed to blood pressure (BP) changes and consequently sustain high-pressure loads over this length, and, consequently, often branch out from large arteries at sharp angles (16). The kidneys and brain are continuously and passively perfused at a high-flow volume throughout systole and diastole, leading to low microvascular resistance. These similar hemodynamic characteristics make the brain and kidney vascular beds vulnerable to fluctuations in BP; thus, both organs are susceptible to microvascular damage (8). Moreover, shared vascular risk factors, such as hypertension, lead to endothelial dysfunction and vascular remodeling, creating a vicious cycle that perpetuates end-organ damage and, in turn, affects local autoregulation. Specifically, CKD narrows the zone of renal autoregulation, which is regulated through the myogenic reflex of the afferent glomerular arteriole and tubule-glomerular feedback (17). Elevated BP variability may further increase the susceptibility of the brain and kidney vasculature to endothelial dysfunction. Current evidence suggests that elevated BP variability is associated with cardiovascular events and death in the CKD population (18–20).

## Cerebral small-vessel disease

Cerebral SVD is the umbrella term used to describe pathologies of vascular structures (small arteries, arterioles, capillaries, small veins, and venules) that supply the brain (21). The consistently identified risk factors for all forms of SVD are advanced age and hypertension, adding to evidence from genetic studies which have shown associations between SVD and hypertension (22, 23). SVD is characterized by a heterogeneous spectrum of histopathological features possibly initiated by endothelial dysfunction, BBB disruption, inflammation, oxidative stress, cerebrovascular reactivity decline, and genetic predisposition (24). The Standards for Reporting Vascular Changes on Neuroimaging (STRIVE) definitions were developed to standardize terms that describe the appearance of sequelae of cerebral SVD, including recent small subcortical infarcts, lacunes, WMHs of presumed vascular origin, PVSs, CMBs, cortical superficial siderosis, and brain atrophy on imaging (25). All forms of SVD have a clinical impact on various conditions such as stroke, cognitive impairment, dementia, and disabilities (motor and gait impairment, urination disorder, and depression) (24).

Shared vascular risk factors, predominantly hypertension and diabetes, either independently or in combination, predispose patients with CKD to simultaneous systemic endothelial impairment (26). Even in early renal impairment, oxidative stress, low-grade inflammation, and reduced nitric oxide availability make the endothelium more vulnerable to slight vascular shifts, which, in turn, compromise the BBB integrity and facilitate infiltration by white blood cells (9, 27). There is an overlap between circulating inflammatory markers, such as C-reactive protein, interleukin-1 (IL)-1, IL-6, and tumor necrosis factor-α in patients with CKD, suggesting a similar course of inflammation in both organs (9, 28).

Although overt uremia is typically recognized when GFR declines to <15 mL/min/1.73 m^2^, it is clear that metabolite accumulation occurs at an earlier stage of CKD (26). Uremic toxins have been reported to directly alter the integrity of vascular endothelial cells and induce BBB disruption and arterial stiffness through increased oxidative stress (9, 27). Indoxyl sulfate decreases cerebral endothelial cell viability *in vitro*, which is associated with a decrease in nitric oxide production and an increase in the production of reactive oxygen species, inducing arterial stiffness (29). The aryl hydrocarbon receptor is the receptor of indoxyl sulfate in endothelial cells and is widely expressed in the central nervous system, such as the hippocampus. Activation of aryl hydrocarbon receptor by indoxyl sulfate also causes BBB disruption, which induces cognitive impairment in rodent models with CKD (30). These findings suggest a pathogenic role for uremic toxins in affecting BBB permeability and promoting SVD development.

Accumulating evidence has indicated that cerebral SVD is commonly observed in patients with renal impairment. A meta-analysis of pooled results from 27 studies (largely cross-sectional) confirmed the independent association between microalbuminuria and SVD, including WMHs, lacunes, CMBs, and PVSs in both the centrum semiovale and basal ganglia (31). These articles provide compelling evidence that albuminuria is a surrogate marker of microvascular disease and may be reflective of systemic vascular endothelial damage (31).

CKD contributes to medial calcification, remodeling, and stiffening of the large arteries. In a population-based study (Atherosclerosis Risk In Communities [ARIC] study), transcranial Doppler measurements revealed an inverse association between the degree of cerebral artery stiffness and CKD (32). When the pulsatility in large-artery disease is compromised, the downstream pressure pulsatility can be readily transmitted into the small vessels of the brain and kidney and is characterized by a low hydrodynamic resistance, resulting in subsequent vascular injury, SVD, and brain atrophy (33, 34). In a retrospective hospital-based study involving post-stroke patients, those with CKD were found to have a significantly higher SVD burden and higher distal intracranial resistance in the anterior cerebral circulation (35). In all, the small and large vessels are likely to exhibit parallel impairments in patients with CKD.

## White matter hyperintensities

The predominant radiological manifestations of SVD are WMHs in the periventricular and deep white matter, with ischemic demyelination, axonal loss, and gliosis, corresponding to the WMHs seen on T2-weighted MRI (36). The prevalence of WMHs of presumed vascular origin increases exponentially with age at any degree of severity, and occurs in 90% of individuals older than 80 years. In addition to aging, WMHs are also more common in individuals with a history of stroke or dementia. In a recent meta-analysis involving 14,000 participants from the general population and those with vascular risk factors, WMHs burden was associated with more than a 2-fold risk of ischemic stroke and a 3-fold higher risk of intracranial hemorrhage than those patients with no or mild WMHs burden (13).

Studies have reported that increased WMHs burden was observed in the CKD population with or without a history of stroke (37–45). As CKD worsens with age due to exposure to vascular risk factors and the cumulative effects of endothelial dysfunction and inflammation, WMHs are generally manifested in patients with CKD, particularly patients undergoing hemodialysis (41). In patients approximately 60 years of age undergoing hemodialysis, WMHs burden is present in >50%, while WMHs was incidentally observed in 11–21% of the age-matched general population (41). Three population-based cross-sectional studies demonstrated that renal impairment (decreased estimated GFR [eGFR] and/or albuminuria) at baseline was independently associated with WMHs burden (37, 38, 40). This association has been replicated in the population-based AGES-Reykjavik study (*n* = 2,671), which considered a longitudinal change in kidney function, and demonstrated participants with an eGFR decline of >3 mL/min/1.73 m^2^/year or incident albuminuria was associated with the progression of WMHs volume (difference [95% confidence interval]:8% [4–12%], 21% [14–29%], respectively) (39). These results may indicate that SVD development could simultaneously progress with renal function decline. In a recent meta-analysis, pooling results from seven prospective cohort studies (*n* = 2,796), systolic BP variability (per 1SD increase) was associated with 1.26 higher odds of the presence or progression of WMHs (46). Any time scale of BP variability (visit-to-visit, day-to-day, hour-to-hour) has contributed to a higher risk of the presence of SVD (46). However, it remains to be elucidated whether this association suggests a target for therapeutic intervention or is the reflection of advanced systemic vascular burden.

## Lacunes

Lacunes are thought to result from the occlusion of penetrating arteries predominantly due to lipohyalinosis or *in situ* microatheroma, which results in focal necrosis in the neural tissue (47). According to the Trial of ORG 10172 in Acute Stroke Treatment (TOAST) criteria, lacunar strokes are small subcortical brain infarcts visible on MRI, <1.5 cm in the axial diameter, and associated with one of the traditional clinical lacunar syndromes (14). STRIVE proposed the term recent lacunar infarcts to define the neuroimaging evidence of recent infarction with an axial diameter of 2 cm in diffusion-weighted imaging MRI sequences in the territory of one perforating arteriole (deep cerebral white matter, basal ganglia, thalamus, or pons), in addition to imaging features or clinical symptoms consistent with a lesion (25). As the lesions are interrelated due to the shared pathogenesis, acute small subcortical infarcts can disappear, remain as WMHs, or form lacunes (25).

Previous studies on the association between renal impairment and lacunar infarcts have been conflicting. Results of a systematic review showed no specific association between renal impairment (decreased eGFR and/or albuminuria) and symptomatic lacunar stroke, but silent lacunar infarction was associated with renal impairment (48). Similarly, in a population-based study of 3,178 patients with acute stroke, a lower frequency of symptomatic lacunar stroke was observed in the CKD population with acute ischemic stroke, and the association between CKD and lacunar stroke was diminished after adjusting for age, sex, and hypertension (49). In the population-based Rotterdam study, a higher albumin-to-creatine ratio or lower cystatin C-based eGFR (eGFR-cystC) was associated with a higher prevalence of asymptomatic lacunes and with WMHs volume (38). Renal impairment was associated with WMHs, CMBs, and PVSs in patients with lacunar infarcts (50, 51). In a longitudinal study, involving 89 patients with lacunar stroke, decreased eGFR was associated with new CMBs progression (52).

## Cerebral microbleeds

CMBs are small (2–10 mm in diameter) round or ovoid hypointense foci with associated blooming with enhanced visibility on MRI sequences sensitive to susceptibility effects (25). Histopathologically, CMBs represent hemosiderin-laden macrophages. The risk factors for CMBs in elderly populations largely differ according to the location of CMBs, suggesting different underlying microangiopathies. Cerebral amyloid angiopathy (CAA) primarily affects the superficial perforating arteries, whereas hypertensive angiopathy mainly affects the deep perforating arteries (25). Consistently identified risk factors for CMBs are advanced age and hypertension. CKD has been associated with an increased prevalence of CMBs. In a single-center study for health screening, moderate to severely decreased eGFR (<60 mL/min/1.73 m^2^) was associated with the presence of CMBs, particularly deep/infratentorial CMBs (53). As mentioned above, in the population-based Rotterdam study with a cross-sectional design, the participants with the highest quartile of albumin to creatine ratio at baseline, but not decreased eGFR, had a higher frequency of CMBs compared to those with the lowest quartile (38). These associations have been replicated in the longitudinal population-based AGES-Reykjavik study, indicating that participants with incident albuminuria had 1.86 higher odds of developing deep CMBs (39). Several small studies found CMBs in up to 35–50% of patients undergoing hemodialysis (54–56). Apart from aging and hypertension, experimental studies suggest that elevated levels of urea may alter the cytoskeleton of endothelial cells and tight junction proteins and may be partly responsible for CMBs (57). Uremic serum potentially disrupts the cultured brain endothelial monolayer due to disarranged actin cytoskeleton and decreased tight junction proteins in the cells (57).

## Perivascular spaces

PVSs are interstitial fluid-filled cavities surrounding the small penetrating vessels and function as the brain drainage system, such as the glymphatic system (58). Cerebral waste clearance via the glymphatic system relies on the convective movement of perivascular cerebrospinal fluid into the parenchymal interstitial fluid space and adequate drainage into the perivenular space (58). Increasing evidence suggests that the topography of PVSs is characteristic of a specific underlying SVD type: (1) when located in the basal ganglia, PVSs are associated with hypertensive arteriolosclerosis, such as arterial stiffening; and (2) PVSs in the centrum semiovale are related to CAA. This highlights the possible mechanisms behind the impaired clearance of vascular β-amyloid, consistent with the role of PVSs as the brain glymphatic system (58). In a single-center study involving 413 patients with a first-ever acute lacunar stroke, proteinuria and eGFR <60 mL/min/1.73 m^2^ were correlated with PVSs severity in both the centrum semiovale and basal ganglia (50). In a single hospital-based study for acute stroke, white patients with CKD had higher odds of severe centrum-semiovale PVSs when comparing patients with and without CKD within racial groups (59). Among patients with CKD, black patients had 2-fold higher odds of severe PVSs in the basal ganglia and centrum semiovale compared to whites and other racial groups (59). In a single hospital-based study for 304 patients with autosomal-dominant polycystic kidney disease (ADPKD), ADPKD was associated with a higher degree of PVSs, but not with the WMHs severity, lacunes, or CMBs, compared to age-, sex-, and eGFR-matched controls, suggesting that ADPKD-associated cilia dysfunction may induce chronic cerebral glymphatic system dysfunction (60).

## Intracranial atherosclerotic stenosis

The systemic arteriosclerotic process in CKD is characterized by structural alterations in the intrinsic stiffness of the media in the aortic wall (8, 61, 62). These alterations occur during the early stages of renal impairment and simultaneously progress to renal function decline, leading to arterial enlargement and wall thickening. Although the mechanism underlying arterial stiffening in CKD has not been fully elucidated, metabolic abnormalities due to renal impairment, such as uremic milieu-induced oxidative and carbonyl stress, and the decreased clearance of pro-inflammatory cytokines may contribute to the pathogenesis of atherosclerosis (9).

Intracranial atherosclerotic stenosis of major cerebral arteries is a common cause of ischemic stroke. Although CKD affects stroke prognosis in large-artery atherosclerotic stroke (63), studies evaluating the prevalence of intracranial atherosclerotic stenosis remain scarce in both population- and hospital-based cohorts. Previous hospital-based studies of Caucasian patients with stroke/transient ischemic attack (TIA) have reported a wide range of prevalence of symptomatic intracranial stenosis, probably reflecting differences in the definition of intracranial stenosis, imaging techniques, inclusion criteria, and completeness of ascertainment. A population-based study of stroke/TIA (Oxford Vascular Study [OXVASC]) showed symptomatic or asymptomatic 50–99% intracranial stenosis in 17.6% of patients, with the highest rates at older ages (64). The prevalence of any intracranial stenosis (50–99%) increased with age from 7.0% at <50 years to 45.1% at ≥90 years (64). A population-based study (ARIC study), involving 1,762 participants (mean age, 76.3 years), found that eGFR-cysC (<60 mL/min/1.73 m^2^) was associated with the presence of intracranial atherosclerotic stenosis on high-resolution vessel-wall MRI (65). Albuminuria (urine albumin-to-creatinine ratio ≥30) was associated with 50–70% intracranial stenosis. In two Chinese population-based studies, decreased eGFR (<45 mL/min/1.73 m^2^) was independently associated with intracranial atherosclerotic stenosis assessed by transcranial Doppler (66, 67). A causal relationship between intracranial atherosclerotic stenosis and CKD was not established because these previous studies were limited by their cross-sectional design.

## Microstructural changes

Diffusion tensor imaging is a molecular MRI technique that allows the measurement of the diffusion of water molecules along the nerve tracts. It can also be used to evaluate the structural integrity of the white matter and is a sensitive marker of microstructural changes in the brain. A population-based study (Rotterdam study), involving 2,726 participants (mean age, 56.6 years), found that a lower eGFR-cysC and higher albumin-to-creatinine ratio were associated with worse global white matter microstructural integrity (68). Microstructural damage, such as decreased white matter integrity, was consistently observed in patients with ESRD, particularly those undergoing long-term hemodialysis. Previous studies reported decreased fractional anisotropy and increased mean diffusivity in patients undergoing hemodialysis compared to age-matched controls, indicating insidious white matter damage (69–73). Hemodialysis-specific circulatory stress, intradialytic BP variation, and direct uremic toxins may contribute to worsened white matter integrity. No longitudinal study has allowed for the determination of causality between hemodialysis and white matter microstructural integrity. Nevertheless, in a study in which progressive WMHs burden was demonstrated in patients undergoing hemodialysis, improvements in cerebral anisotropic diffusion and CBF were noted in the post-transplantation period, suggesting possible reversibility (74). This result supports the hypothesis that CKD may accelerate covert white matter damage independent of vascular risk factors.

## Brain atrophy

Imaging studies show a consistent positive association between brain atrophy and renal impairment, particularly in patients with ESRD undergoing hemodialysis (75–78) although inconsistent results were observed in the early stages of CKD (79–82). In a cross-sectional study of medical check-up centers comprising 1,215 participants, albuminuria contributed to cortical thinning, predominantly in the frontal and occipital regions (83). The study also suggested that albuminuria was associated with frontal lobe atrophy partially mediated by WMHs burden [83. It is hypothesized that systemic endothelial dysfunction accompanied with albuminuria occurs in the brain, resulting in the extravasation of serum proteins into the brain extracellular spaces and causing brain injury (84). Moreover, brain atrophy could partly occur based on the severity of SVD, which is prominently observed in patients with CKD (85). In patients undergoing hemodialysis, intradialytic hypotension may be involved in brain atrophy. Progression in frontal atrophy, as assessed by MRI, was found to be inversely correlated with the number of intradialytic hypotensive episodes in a longitudinal study (86). In addition, a cross-sectional study revealed that patients with CKD had a lower hippocampal volume and smaller cortical thickness than those in matched controls, providing evidence of a potential link between Alzheimer's disease-related pathology and kidney function, while the mechanisms of hippocampal atrophy in the CKD population are largely unknown (87, 88).

## Cerebral blood flow

CKD is associated with a dysfunctional BBB due to endothelial inflammation and vascular remodeling, which can impair the regulation of local CBF. Impaired autoregulation can lead to increased pressure across the capillary bed, which could result in capillary damage and increased BBB permeability (89). Hemodialysis can induce a transient decline in CBF. Cerebral arterial mean flow velocity has been shown to significantly decline during hemodialysis, and this intradialytic hemodynamic instability could cause transient cerebral stunning (90). Two prospective studies demonstrated that hemodialysis induces decreased intradialytic cerebral perfusion, partly due to intradialytic hypotension (91, 92). In a study of the acute effect of conventional hemodialysis on CBF, measured by positron emission tomography-computed tomography, global CBF declined significantly by 10 ± 15% (92). In contrast, previous hospital-based cohort studies assessed CBF with SPECT or MRI (arterial-spin labeling or phase contrast imaging) in patients undergoing hemodialysis and reported higher CBF compared to patients with normal kidney function (93–97). Regarding patients without hemodialysis, in a large cohort of nondiabetic hypertensive adults in early CKD stages, decreased eGFR (<45 vs. ≥90 mL/min/1.73 m^2^) was associated with a higher total CBF as assessed by arterial spin labeling, and albuminuria was associated with a large WMHs volume (98). However, in the Rotterdam study which excluded patients with ESRD, a cross-sectional analysis of 2,645 participants demonstrated that decreased eGFR was independently associated with lower CBF measured by MRI (99). These contradictory findings are possibly due to the differences in the method of assessing CBF or in patents characteristics such as the stages of CKD. Moreover, it remains unclear whether these CBF changes subsequently lead to cerebral structural changes.

## Discussion

**Figure 1:**
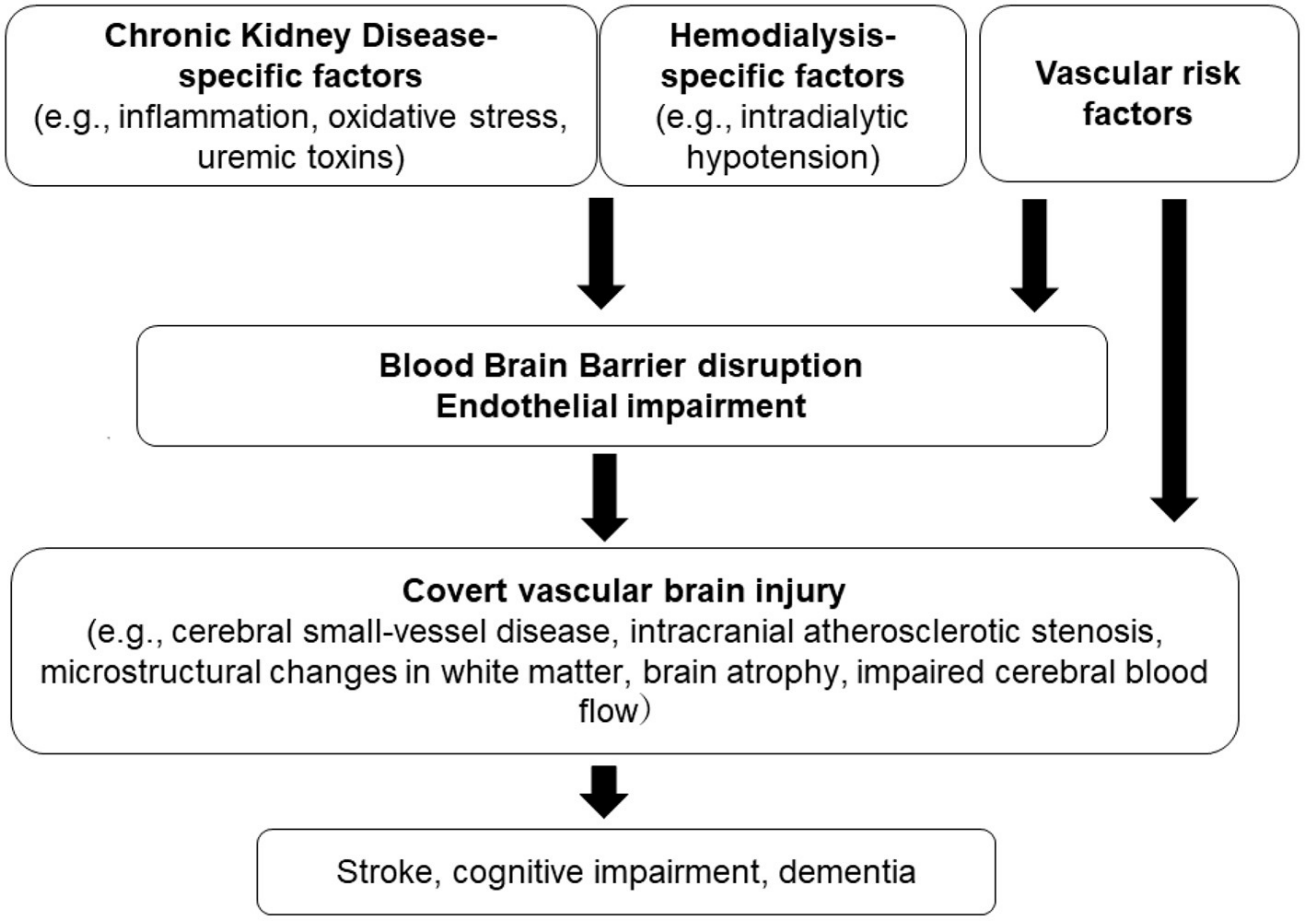
Proposed pathophysiology of chronic kidney disease–related covert brain injury.

We briefly described the association between covert vascular brain injury and CKD. This review includes an up-to-date discussion of imaging findings in patients with CKD, which may provide important insights into the early stages of stroke and dementia. Our narrative review has limitations, as it did not involve quality assessment of the included study reports. Overall, results from the literature collectively indicate that CKD is largely associated with structural brain abnormalities. There is a multifactorial mechanism underlying these brain injuries in the setting of CKD (Figure 1). The kidney-brain association appears to represent greater impairment in kidney function, which could lead to more severe SVD and brain atrophy.

Recent experiments have highlighted that the direct toxicity of uremic toxins, such as the indoxyl sulfate-aryl hydrocarbon receptor pathway, may play an important role in BBB disruption and subsequent cognitive impairment in CKD (30, 57). A recent Mendelian randomization study suggested that renal impairment assessed by higher urine albumin-to-creatinine ratio and decreased eGFR are causally involved in large-artery stroke and SVD (i.e., small-vessel stroke, WMHs, and intracerebral hemorrhage), emphasizing the shared common genetic mechanisms with CKD (100). However, evidence regarding mechanistic pathways to demonstrate the development of these imaging findings is still lacking, since most results are derived from observational studies with a cross-sectional design. The amount and quality of evidence have been limited, especially in advanced CKD, including patients undergoing hemodialysis, due to the small sample sizes.

Apart from kidney transplantation, there is no direct evidence to suggest that any intervention prevents or reduces brain structural abnormalities in the CKD population. In the context of SVD, given both organs are common targets of vascular risk factors, we can speculate that people with CKD could benefit from more intensive vascular risk reduction, with a particular focus on hypertension and diabetes. There is current evidence indicating that intensive BP lowering could be associated with less WMHs progression in hypertensive patients (101, 102). In contrast, there is no evidence for glucose control in the absence of diabetes to prevent SVD progression (102). The recently published ESOC guidelines recommend patients with SVD and hypertension to have their BP well-controlled for the management of SVD with low quality of evidence (103). However, little to no data are currently available on the impact of intensive BP lowering on SVD in patients with CKD. There have been concerns that intensive BP treatment results in a greater risk of acute kidney injury inferred to reflect hemodynamic changes in kidney perfusion rather than true kidney function loss (104, 105). Considering that the pathophysiology of SVD remains incompletely understood and the evidence on the benefits in SVD progression is limited, there is still a research gap in elucidating how additional mechanisms contribute to the development and deterioration of SVD in general, as well as in the CKD population. Thus, additional prospective population-based studies with larger samples, across all CKD stages, and longer follow-up periods are needed to investigate the impact of CKD on SVD. Further experimental studies elucidating the observed association between CKD and SVD are required to identify molecular mechanisms that may enable the development of novel therapeutic approaches beyond the management of vascular risk factors.

## Conclusion

Patients with CKD consistently show a high prevalence of covert vascular brain injuries, such as SVD, brain atrophy, intracranial artery stenosis, microstructural changes, and impaired CBF. These brain injuries, especially endothelial impairment, and BBB disruption are supported by rodent models pointing to CKD-specific causes such as the direct effect of uremic toxicity. Genetic variants that predispose patients to CKD have also been linked to large artery stroke and SVD. However, there is still a dearth of evidence regarding the mechanisms underlying these brain injuries in patients with CKD. Studies are needed for the CKD population to focus on how to prevent the development and progression of these brain injuries, which may be a potential strategy to protect against stroke, vascular cognitive impairment, or dementia.

## Author contributions

Both authors listed have made a substantial, direct, and intellectual contribution to the work and approved it for publication.

## Funding

Supported by a grant from Japan Agency for Medical Research and Development (AMED: JP21lk0201094 and JP21lk0201109).

## Conflict of interest

The authors declare that the research was conducted in the absence of any commercial or financial relationships that could be construed as a potential conflict of interest.

## Publisher's note

All claims expressed in this article are solely those of the authors and do not necessarily represent those of their affiliated organizations, or those of the publisher, the editors and the reviewers. Any product that may be evaluated in this article, or claim that may be made by its manufacturer, is not guaranteed or endorsed by the publisher.

## Abbreviations

ADPKD: autosomal dominant polycystic kidney disease
BBB: blood-brain barrier
CBF: cerebral blood flow
CKD: chronic kidney disease
CMBs: cerebral microbleeds
eGFR: estimated glomerular filtration rate
ESRD: end-stage renal disease
PVSs: perivascular space
GFR: glomerular filtration rate
IL: interleukin
MRI: magnetic resonance imaging
STRIVE: Standards for Reporting Vascular Changes on Neuroimaging
SVD: small-vessel disease
WMHs: white matter hyperintensities.
